# Musculoskeletal Disorders, Pain Medication, and in-Hospital Mortality among Patients with COVID-19 in South Korea: A Population-Based Cohort Study

**DOI:** 10.3390/ijerph18136804

**Published:** 2021-06-24

**Authors:** Tak-Kyu Oh, In-Ae Song, Joon Lee, Woosik Eom, Young-Tae Jeon

**Affiliations:** 1 Department of Anesthesiology and Pain Medicine, Seoul National University Bundang Hospital, Seongnam 13620, Korea; airohtak@hotmail.com (T.-K.O.); songoficu@outlook.kr (I.-A.S.); 2 Department of Anesthesiology and Pain Medicine, National Cancer Center, Goyang 10408, Korea; leejoon.com@gmail.com; 3 Department of Anesthesiology and Pain Medicine, College of Medicine, Seoul National University, Seoul 03080, Korea

**Keywords:** analgesics, opioid, musculoskeletal diseases, pain, population

## Abstract

We aimed to investigate whether comorbid musculoskeletal disorders (MSD)s and pain medication use was associated with in-hospital mortality among patients with coronavirus disease 2019 (COVID-19). Adult patients (≥20 years old) with a positive COVID-19 diagnosis until 5 June 2020 were included in this study, based on the National Health Insurance COVID-19 database in South Korea. MSDs included osteoarthritis, neck pain, lower back pain, rheumatoid arthritis, and others, while pain medication included paracetamol, gabapentin, pregabalin, glucocorticoid, nonsteroidal anti-inflammatory drugs (NSAIDs), opioids (strong and weak opioids), and benzodiazepine. Primary endpoint was in-hospital mortality. A total of 7713 patients with COVID-19 were included, and in-hospital mortality was observed in 248 (3.2%) patients. In multivariate logistic regression analysis, no MSDs (*p* > 0.05) were significantly associated with in-hospital mortality. However, in-hospital mortality was 12.73 times higher in users of strong opioids (odds ratio: 12.73, 95% confidence interval: 2.44–16.64; *p* = 0.002), while use of paracetamol (*p* = 0.973), gabapentin or pregabalin (*p* = 0.424), glucocorticoid (*p* = 0.673), NSAIDs (*p* = 0.979), weak opioids (*p* = 0.876), and benzodiazepine (*p* = 0.324) was not associated with in-hospital mortality. In South Korea, underlying MSDs were not associated with increased in-hospital mortality among patients with COVID-19. However, use of strong opioids was significantly associated with increased in-hospital mortality among the patients.

## 1. Introduction

The coronavirus disease 2019 (COVID-2019) was declared a pandemic by the World Health Organization on 11 March 2020 [1]. As of 6 April 2021, the global toll of COVID-19 cases was 131,699,808, and the number of deaths was 2,859,642 [2]. Although vaccine administration began on 8 December 2020 [3,4], limitations associated with the volume of vaccine production and the rapidity of administration hampered the attainment of herd immunity against COVID-19. Thus, the COVID-19 crisis remains the most serious global health issue in 2021.

Immune-mediated inflammation is the main mechanism of COVID-19 infection, known to be an important prognostic factor among patients who contracted the disease [5,6]. Further, due to impaired immune defences both from underlying disease and treatment, immunocompromised patients with respiratory virus infection are at risk of a more severe infection [7,8]. Immunocompromised patients with cancer and organ transplantation are known to have worse COVID-19 prognosis; however, the prognosis of other immunocompromised patients treated with biologics for chronic disease or those with acquired immune deficiency syndrome remains controversial [9].

As immune system dysregulation may play a role in chronic pain, and individuals with chronic musculoskeletal pain reportedly had abnormal immune responses [10], it might affect the prognosis of patients with COVID-19. Furthermore, pain medications such as opioids and glucocorticoids are known to cause immunosuppression if prescribed long-term [11,12,13]. Thus, long-term prescription of opioids or glucocorticoids is associated with increased mortality in a South Korean population [14,15]. Based on evidence of the immunosuppressive effects of long-term opioid use, the prognosis of long-term opioid users might worsen after COVID-19 [16]. However, information regarding the impact of chronic musculoskeletal disorders (MSD) or the use of pain medication on the prognosis of patients with COVID-19 remains unidentified.

We hypothesized that comorbid MSDs and use of pain medication might increase in-hospital mortality among patients with COVID-19. Therefore, we aimed to investigate this hypothesis in South Korea.

## 2. Materials and Methods

### 2.1. Study Design and Ethical Statement

This population-based cohort study was conducted according to the Reporting of Observational Studies in Epidemiology guidelines [17]. Deliberation of the study protocol was exempted by the Institutional Review Board (IRB) of Seoul National University Bundang Hospital (X-2004-604-905) and the National Health Insurance Service (NHIS) data sharing service (NHIS-2020-1-424). Informed consent was waived by the IRB, as the analyses were performed retrospectively using anonymized data derived from the South Korean NHIS database.

### 2.2. NHIS-COVID-19 Cohort Database and Study Population

The NHIS-COVID-19 cohort database was created in cooperation between The Korea Disease Control and Prevention Agency (KDCPA) and NHIS for medical research associated with the COVID-19 crisis in South Korea. The KDCPA provides data of individuals who were diagnosed with COVID-19 in the polymerase chain reaction (PCR) test from 1 January to 4 June 2020. Therefore, the database contains information on all patients with COVID-19 regardless of disease severity, which includes demographic information, date of diagnosis, and treatments results. However, the data from patients who were receiving ongoing in-hospital treatment were not included, as the treatment results were not determined at that time. As a sole public insurance system, all disease diagnoses of patients with COVID-19 from 2015 to 2020 were registered in the database using International Classification of Diseases (ICD)-10 codes. Additionally, all prescription information of drugs and/or procedures from 2015 to 2020 was also included.

For this study, we included individuals who received a positive COVID-19 diagnosis between 1 January 2020 to 4 June 2020 in South Korea, and individuals <20 years old were excluded from the analysis. In South Korea, patients with COVID-19 are admitted to the hospital only if they have severe symptoms such as pneumonia. However, if they have no symptoms or only mild symptoms, they are asked to isolate and are closely monitored in specific centres managed by the government.

### 2.3. Exposure Variables: Musculoskeletal Disease and Pain Medication

Osteoarthritis, neck pain, low back pain, rheumatoid arthritis, and other MSDs were defined as MSD in this study. A previous MSD diagnosis was evaluated using the ICD-10 codes of the NHIS database from 2015 to 2019 (data shown in Table S1). Additionally, instances of cancer, metastatic solid tumours, and major depressive symptoms were also included in the analysis, as they are closely related to the use of pain medication.

For pain-related medications, prescription data from 2019 to 2020 were extracted for drugs including paracetamol, gabapentin, pregabalin, glucocorticoids, nonsteroid anti-inflammatory drugs (NSAIDs), opioids (strong and weak opioids), and benzodiazepines. Considering the variability in opioid potency [18], codeine, dihydrocodeine, hydrocodone, and tramadol were categorized as weak opioids, and all others (e.g., fentanyl, morphine, oxycodone, hydromorphone, and methadone) were categorized as strong opioids. Pain medication users were defined as those prescribed a continuous drug supply over a period of ≥90 days from 2019 until the date of COVID-19 diagnosis. For example, an individual who was prescribed strong opioids, benzodiazepines, and gabapentin over a period of ≥90 days was classified as a user of strong opioids, benzodiazepines, and gabapentin in this study. Only patients who continuously took the drug within 1 month before the date of COVID-19 diagnosis by a PCR test were considered pain medication users. In South Korea, opioids, benzodiazepines, and gabapentin need to be prescribed by physicians; thus, there were no missing prescription data for these drugs. However, paracetamol and NSAIDs are available in convenience stores or the market without a prescription.

### 2.4. Endpoints

The primary endpoint of this study was in-hospital mortality among patients with COVID-19 in the NHIS-COVID-19 database, evaluated from 1 January 2020 to 27 August 2020. In-hospital mortality was defined as COVID-19-related deaths among patients with COVID-19. If a patient died after end of isolation, as the virus was no longer detected in the PCR test, it was not considered a COVID-related death.

### 2.5. Covariates

Some information was extracted as confounders for this study, which included demographic characteristics such as age and sex. The NHIS classified age into the following seven groups (categorical variable) to maintain anonymity in the database: 20–29, 30–39, 40–49, 50–59, 60–69, 70–79, and ≥80 years old groups. Annual income level in 2020 and place of residence were collected and used as confounders to reflect socio-economic status of the study participants. The place of residence was divided into the following five groups based on the COVID-19 cases until 4 June 2020: Seoul, Gyeonggi-do, Daegu, Gyeongsangbuk-do, and other areas. Comorbid status among the patients was reflected based on the underlying disability and the Charlson comorbidity index, calculated using the registered ICD-10 diagnostic codes (Table S2), from 1 January 2015 to 31 December 2019. In South Korea, the social welfare system requires all individuals with disabilities to be registered in the NHIS database to get various benefits. The disabilities were divided into six grades according to the severity, and we used two severity groups (1–3: severe disability; 4–6: mild-to-moderate disability).

### 2.6. Statistical Analysis

Baseline characteristics of the patients with COVID-19 are presented as mean values with standard deviations for continuous variables (Charlson comorbidity index) and numbers with percentages for categorical variables (all other variables). First, we performed univariate logistic regression analysis to identify the individual association of all variables with in-hospital mortality among patients with COVID-19 in South Korea. We then constructed a multivariate logistic regression model to identify the independent associations of all variables with in-hospital mortality among patients with COVID-19 in South Korea. In the multivariate model, all covariates were included for multivariate adjustment. No multicollinearity was observed in any multivariate model of the entire cohort, with a variance inflation factor <2.0. The results of the logistic regression analyses are presented as odds ratios (ORs) with 95% confidence intervals (CIs). R software (version 4.0.3; R Foundation for Statistical Computing, Vienna, Austria) and SAS software version 9.4 (SAS Institute Inc., Cary, NC, USA) were used for all analyses, and *p* < 0.05 was considered statistically significant.

## 3. Results

### 3.1. Study Population

**Figure 1:**
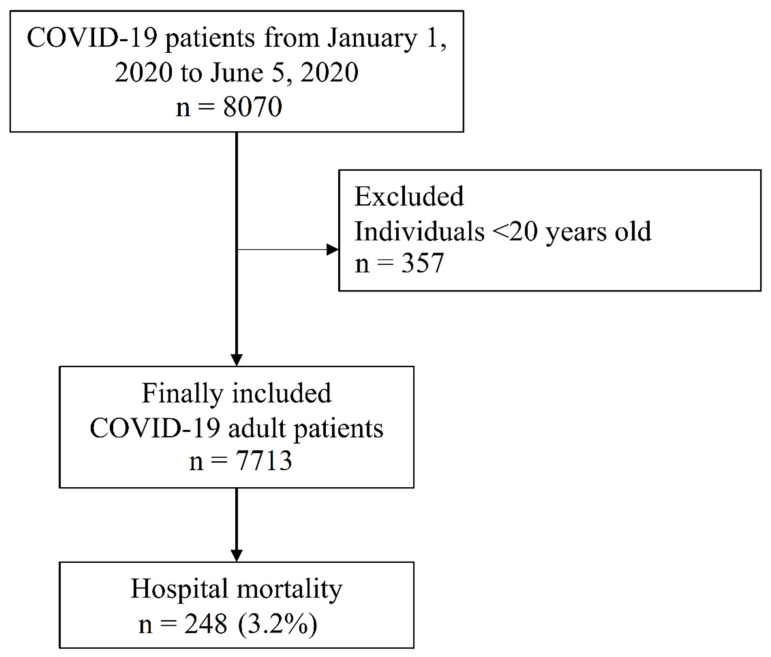
Flow chart depicting selection of patients with COVID-19 in South Korea. COVID-19, coronavirus disease 2019.

The NHIS-COVID-19 database included 8070 patients with COVID-19. Among them, 357 individuals aged <20 years old were excluded from the analysis. Finally, 7713 patients with COVID-19 were included. From these patients, 216 (3.0%) were admitted to the intensive care unit, 127 (1.8%) received mechanical ventilation, and 21 (0.3%) received extracorporeal membrane oxygenation. Finally, in-hospital mortality was observed in 248 (3.2%) patients (Figure 1). Baseline characteristics of the patients with COVID-19 are presented in Table 1. There were 3641 (47.2%) patients with osteoarthritis, 1971 (25.6%) with neck pain, 4836 (62.7%) with lower back pain, 739 (9.6%) with rheumatoid arthritis, and 4908 (63.6%) had other MSDs. Among pain medications, 58 (0.8%) patients used paracetamol, 148 (1.9%) used gabapentin or pregabalin, 63 (0.8%) used glucocorticoid, one (0.0%) used NSAIDs, nine (0.1%) used strong opioids, 240 (3.1%) used weak opioids, and 259 (3.4%) used benzodiazepine. The nine patients with chronic strong opioid use included three patients with cancer and six patients with chronic MSD.

### 3.2. In-Hospital Mortality among Patients with COVID-19

Table 2 and Table 3 show the results of univariate and multivariate logistic regression analysis for in-hospital mortality among patients with COVID-19. In the multivariate model, osteoarthritis (OR: 1.11, 95% CI: 0.75–1.65; *p* = 0.589), neck pain (OR: 0.74, 95% CI: 0.52–1.05; *p* = 0.095), lower back pain (OR: 0.73, 95% CI: 0.49–1.10; *p* = 0.133), rheumatoid arthritis (OR: 0.78, 95% CI: 0.50–1.23; *p* = 0.291), and other MSDs (OR: 0.73, 95% CI: 0.49–1.09; *p* = 0.129) were not associated with in-hospital mortality.

However, regarding pain medications, in-hospital mortality was 12.73 times higher for users of strong opioids (OR: 12.73, 95% CI: 2.44–16.64; *p* = 0.002). In contrast, compared with that for other patients, in-hospital mortality was not significantly high for users of paracetamol (OR: 0.98, 95% CI: 0.37–2.62; *p* = 0.973), gabapentin or pregabalin (OR: 0.77, 95% CI: 0.41–1.45; *p* = 0.424), glucocorticoid (OR: 1.26, 95% CI: 0.43–3.63; *p* = 0.673), NSAIDs (OR: 0.00, 95% CI: 0.00-; *p* = 0.979), weak opioids (OR: 0.96, 95% CI: 0.55–1.67; *p* = 0.876), and benzodiazepines (OR: 0.77, 95% CI: 0.46–1.29; *p* = 0.324).

## 4. Discussion

This population-based cohort study using the NHIS-COVID-19 database showed that MSDs were not associated with increased in-hospital mortality in patients with COVID-19 compared to those without MSDs. Further, use of strong opioids was associated with higher in-hospital mortality among patients with COVID-19 compared to non-users, while use of other pain medications (paracetamol, gabapentin, pregabalin, glucocorticoids, NSAIDs, weak opioids, and benzodiazepines) was not significantly associated with in-hospital mortality. Our results suggest that comorbid MSDs and use of pain medication, except for strong opioids, do not worsen the prognosis of patients with COVID-19, at least in South Korea.

Although most pain medications were not associated with in-hospital mortality, strong opioids showed a significant association with in-hospital mortality. This could be attributed to the immunosuppression caused by chronic strong opioid use [16]. However, there might be an indication bias in terms of long-term opioid prescription [19], because long-term strong opioid users are more commonly elderly individuals with more comorbidities, who are more likely to develop severe COVID-19 and die. Thus, in addition to long-term strong opioid use, the patients’ age and comorbid status possibly affected the results. It should also be noted that there were only nine long-term strong opioid users, and their OR was 12.73 (95% CI: 2.44–16.44). Thus, the number of strong opioid users was not adequate for estimating OR for in-hospital mortality, and this sparsity bias possibly affected the results [20]. Accordingly, the result regarding the association of strong opioid use with in-hospital mortality should be interpreted with caution and validated in further studies with larger samples.

The association between opioid use and increased mortality was reported in the United States [21,22], the United Kingdom [23], and South Korea [14]. A recent study reported that opioid use was closely related to the growing trend of infectious diseases in the United States, and opioid use created a converging public health crisis with a significant combined impact on morbidity and mortality with regard to infection [24]. Additionally, a recent nationwide cohort study in the United States on hospitalized patients with serious infection showed that underlying opioid use disorder was linked to poorer hospital outcomes [25]. Another prospective cohort study reported the association between higher all-cause mortality and long-term opioid prescription among both human immunodeficiency virus-infected and uninfected individuals [26].

With respect to the relationship between opioid use and COVID-19 outcomes, the biological plausibility of a correlation between opioid use disorder and worse COVID-19 outcomes was suggested in July 2020 [27]. In September 2020, Wang et al. reported that substance disorders, including opioid use disorder, were associated with increased mortality among patients with COVID-19 in the United States [28]. However, there were some differences between the study by Wang et al. and ours. First, Wang et al. used just age, sex, race, and insurance type for adjustment as covariates, whereas we included numerous covariates that might be closely related to opioid use, such as Charlson comorbidity index, MSD, depression, and various pain medications, including benzodiazepines. Second, we focused on long-term opioid users instead of individuals with opioid use disorder, suggesting that the users of strong opioids in our study were appropriately prescribed medication by physicians for pain management. Considering all of the above, our results should be interpreted carefully. Furthermore, in addition to individuals with opioid use disorder, the risk of mortality in normal strong opioid users might be increased among patients with COVID-19. Recent reports emphasized the importance of prevention of opioid use disorder during the COVID-19 pandemic, as primary care physicians had to prescribe larger opioid doses for longer periods or prescribe remotely to maintain physical distancing [29,30]. Therefore, the results of our study also suggest that prevention and management among opioid users are important during the COVID-19 pandemic.

The impact of MSDs on in-hospital mortality among patients with COVID-19 remains unclear. In a single-centre retrospective cohort study, Fredi et al. reported that older age and comorbidities are associated with poorer outcomes among patients with rheumatic and musculoskeletal diseases [31]. In another prospective cohort study, COVID-19 outcomes were worse in patients with underlying inflammatory arthritis who were receiving glucocorticoids [32]. However, no previous studies evaluated whether MSDs might be a risk factor for poorer prognosis among patients with COVID-19 compared to the general population [31,32]. MSDs are critical conditions for human function, impairing mobility, dexterity, and the ability to work and actively participate in all aspects of life [33]. Based on our initial findings on the associations of MSDs with in-hospital mortality among patients with COVID-19 in South Korea and the aforementioned studies, the impact of MSDs on COVID-19 prognosis should be further evaluated.

This study has some limitations. First, some important variables, including body mass index and lifestyle factors such as history of smoking and alcohol consumption, were not included in the analysis, as they were not available in the NHIS-COVID-19 database. Second, we defined comorbidities using ICD-10 codes. However, the diseases specified by the ICD-10 codes may differ from the actual underlying diseases in our study population. Third, because NSAIDs and paracetamol are available in convenience stores or the market without a prescription in South Korea, there could be missing cases involving use of these drugs in our database, and the association of NSAIDs and paracetamol use with in-hospital mortality should be interpretated with caution. Fourth, despite our recent cohort study reporting the prevalence of users of strong opioids in 2015 as 0.24% [14], the proportion of users of strong opioids in our study was as low as 0.1% (9 of 7713). Due to this very low proportion in our study, the validity of our results might be controversial. Finally, as mentioned above, the indication bias and the sparsity data bias possibly affected the results regarding the association of strong opioid use with in-hospital mortality.

## 5. Conclusions

Conclusively, in South Korea, underlying MSDs were not associated with increased in-hospital mortality among patients with COVID-19. However, strong opioid use was associated with higher in-hospital mortality among patients with COVID-19, whereas use of other pain medications (paracetamol, gabapentin, pregabalin, glucocorticoids, NSAIDs, weak opioid, and benzodiazepine) did not show a significant association with in-hospital mortality.

## Figures and Tables

**Table 1:** Baseline characteristic of COVID-19 patients in South Korea (*n* = 7713).

Variable	Number (%)	Mean (SD)
Sex, male	3048 (39.5%)	
Age		
20–29	2057 (26.7%)	
30–39	832 (10.8%)	
40–49	1036 (13.4%)	
50–59	1567 (20.3%)	
60–69	1199 (15.5%)	
70–79	617 (8.0%)	
≥80	405 (5.3%)	
Income in qurtile		
Q1 (Lowest)	2439 (31.6%)	
Q2	1445 (18.7%)	
Q3	1577 (20.4%)	
Q4 (Highest)	2135 (27.7%)	
unknown	117 (1.5%)	
Underlying disability		
Mild degree	318 (4.1%)	
Moderate to severe degree	293 (3.8%)	
Residence		
Seoul	510 (6.6%)	
Gyeonggi-do	431 (5.6%)	
Daegu	5036 (65.3%)	
Gyeongsangbukdo	933 (12.1%)	
Other area	803 (10.4%)	
Charlson comorbidity index		2.7 (2.7)
Any cancer	602 (7.8%)	
	70 (0.9%)	
Metastatic solid tumour	3641 (47.2%)	
Neck pain	1971 (25.6%)	
Lower back pain	4836 (62.7%)	
Rheumatoid arthritis	739 (9.6%)	
Other musculoskeletal disease	4908 (63.6%)	
Major depressive disorder	1450 (18.8)	
Pain medication		
Paracetamol	58 (0.8%)	
Gabapentin or pregabalin	148 (1.9%)	
Glucocorticoid	63 (0.8%)	
NSAIDs	1 (0.0%)	
Strong opoioid	9 (0.1%)	
Weak opioid	240 (3.1%)	
Benzodiazepine	259 (3.4%)	

Presented as mean with standard deviation or number with percentage. COVID-19, coronavirus disease 2019; SD, standard deviation; NSAIDs, non-steroidal anti-inflammatory drugs.

**Table 2:** Univariate logistic regression analysis for in-hospital mortality among COVID-19 patients in South Korea.

Variable	Univariate Analysis	*p*-Value
	OR (95% CI)	
Age, 10 year increase	3.56 (3.13, 4.05)	<0.001
Sex, male (vs. female)	1.90 (1.47, 2.45)	<0.001
Annual income level in 2020		
Q1 (Lowest)	1	
Q2	0.63 (0.42, 0.95)	0.026
Q3	0.87 (0.61, 1.25)	0.446
Q4 (Highest)	1.08 (0.79, 1.47)	0.636
Unknown	0.73 (0.23, 2.34)	0.595
Residence at 2010		
Seoul	1	
Gyeonggi-do	5.19 (1.73, 15.56)	0.003
Daegu	3.94 (1.45, 10.67)	0.007
Gyeongsangbukdo	7.92 (2.86, 21.99)	<0.001
Other area	3.23 (1.10, 9.51)	0.033
Underlying disability		
Mild degree (vs. no disability)	5.05 (3.46, 7.37)	<0.001
Moderate to severe (vs. no disability)	5.72 (3.93, 8.33)	<0.001
Charlson comorbidity index, 1 point increase	1.40 (1.35, 1.45)	<0.001
Any cancer	3.70 (2.71, 5.04)	<0.001
Metastatic solid tumour	4.57 (2.24, 9.31)	<0.001
Major depressive disorder	4.18 (3.23, 5.40)	<0.001
Osteoarthritis	3.26 (2.45, 4.35)	<0.001
Neck pain	0.99 (0.74, 1.33)	0.956
Lower back pain	1.94 (1.44, 2.61)	<0.001
Rheumatoid arthritis	1.69 (1.18, 2.42)	0.004
Other musculoskeletal disease	1.63 (1.23, 2.18)	<0.001
Pain medication		
Paracetamol	0.99 (0.35, 2.52)	0.921
Gabapentin or pregabalin	4.42 (2.65, 7.35)	<0.001
Glucocorticoid	2.63 (1.04, 6.61)	0.040
NSAIDs	0.00 (0.00-)	0.978
Strong opoioid	38.38 (10.24, 143.81)	<0.001
Weak opioid	3.06 (1.92, 4.88)	<0.001
Benzodiazepine	3.64 (2.37, 5.57)	<0.001

COVID-19, coronavirus disease 2019; OR, odds ratio; CI, confidence interval; NSAIDs, non-steroidal anti-inflammatory drugs.

**Table 3:** Multivariate logistic regression analysis for in-hospital mortality among COVID-19 patients in South Korea.

Variable	Multivariate Model	*p*-Value
	OR (95% CI)	
**Age, 10 year increase**	3.21 (2.75, 3.75)	<0.001
**Sex, male (vs. female)**	2.13 (1.57, 2.88)	<0.001
Annual income level in 2020		
Q1 (Lowest)	1	
Q2	1.11 (0.69, 1.79)	0.666
Q3	1.01 (0.67, 1.54)	0.949
Q4 (Highest)	0.86 (0.59, 1.24)	0.411
Unknown	0.82 (0.22, 3.10)	0.773
Residence at 2010		
Seoul	1	
Gyeonggi-do	2.82 (0.83, 9.54)	0.096
Daegu	1.76 (0.59, 5.20)	0.308
Gyeongsangbookdo	2.08 (0.68, 6.37)	0.199
Other area	2.34 (0.72, 7.61)	0.157
Underlying disability		
Mild degree (vs. no disability)	0.91 (0.59, 1.40)	0.653
**Moderate to severe (vs. no disability)**	2.88 (1.81, 4.60)	<0.001
**Charlson comorbidity index, 1 point increase**	1.19 (1.12, 1.26)	<0.001
Any cancer	1.00 (0.66, 1.52)	0.986
Metastatic solid tumour	0.79 (0.27, 2.31)	0.665
Major depressive disorder	1.28 (0.93, 1.76)	0.125
Osteoarthritis	1.11 (0.75, 1.65)	0.589
Neck pain	0.74 (0.52, 1.05)	0.095
Lower back pain	0.73 (0.49, 1.10)	0.133
Rheumatoid arthritis	0.78 (0.50, 1.23)	0.291
Other musculoskeletal disease	0.73 (0.49, 1.09)	0.129
Pain medication		
Paracetamol	0.98 (0.37, 2.62)	0.973
Gabapentin or pregabalin	0.77 (0.41, 1.45)	0.424
Glucocorticoid	1.26 (0.43, 3.63)	0.673
NSAIDs	0.00 (0.00-)	0.979
**Strong opoioid**	12.73 (2.44, 16.64)	0.002
Weak opioid	0.96 (0.55, 1.67)	0.876
Benzodiazepine	0.77 (0.46, 1.29)	0.324

Variables with statistical significance are presented in bold font. COVID-19, coronavirus disease-2019; OR, odds ratio; CI, confidence interval; NSAIDs, non-steroidal anti-inflammatory drugs.

## Data Availability

Data will be available upon reasonable request to the corresponding author.

## Supplementary Materials

The following are available online at https://www.mdpi.com/article/10.3390/ijerph18136804/s1, Table S1: ICD-10 codes and Table S2: The ICD-10 codes used by comorbidity to compute the Charlson comorbidity index.
